# Strategy for Making a Superior Quenchbody to Proteins: Effect of the Fluorophore Position

**DOI:** 10.3390/s140713285

**Published:** 2014-07-23

**Authors:** Hee-Jin Jeong, Hiroshi Ueda

**Affiliations:** Chemical Resources Laboratory, Tokyo Institute of Technology, 4259-R1-18 Nagatsuta-cho, Midori-ku, Yokohama 226-8503, Japan; E-Mail: heejin@pe.res.titech.ac.jp

**Keywords:** Quenchbody, single chain antibody, fluorescence quenching, fluorescent biosensor, hen egg lysozyme, photoinduced electron transfer, *in vitro* translation

## Abstract

Antibody-based sensors have made outstanding contributions to the fields of molecular biology and biotechnology. Our group recently developed a novel powerful fluorescent immunosensor strategy named Quenchbody (Q-body), which has been applied to the detection of a range of antigens in a rapid, simple, and sensitive manner. However, there were some Q-bodies whose fluorescence response was limited, especially for detecting protein antigens. With the aim of improving this issue, here we made twelve types of Q-bodies incorporated with different number and position of TAMRA fluorophore in the single chain Fv of HyHEL-10, an anti-hen egg lysozyme antibody, as a model. By measuring the fluorescence intensity and its antigen dependency, it was revealed that V_L_-V_H_ type Q-bodies labeled at a non-CDR loop region of the V_L_ shows the highest fluorescence response. This position locates close to the quenching Trp35 in V_L_, while it is far from Trp residues in the bound antigen. This result clearly suggests the importance of dye position to maximize the fluorescence quenching and antigen-dependent de-quenching. The discovery may open a way to make many other Q-bodies with superior response.

## 1. Introduction

Antibody-based biosensors represent practical and precise tools for detecting antigens with their high selectivity, specificity and immediacy. Those immunosensors have been increasingly demanded in the fields of scientific and analytical applications, and have been widely used in molecular biology and biomedical research, as well as in clinical diagnosis [1,2]. Among them, fluorescent immunosensors, which are made by conjugation of fluorescent probes to antibodies, have merits such as no need of additional reagents and availability of sensitivity without need of long incubation. Fluorescence is an optical signal that allows one to detect molecular interactions with great sensitivity. The transduction is based on a variation of the fluorescence properties of the biosensor when it interacts with its analyte [3]. Although intrinsic protein fluorescence can be used to study molecular interactions in purified experimental systems, extrinsic fluorescence is normally preferable to monitor specific interactions in complex media, without interference from other protein components [4].

For example, Förster resonance energy transfer (FRET), which occurs when two fluorophores, donor and acceptor, are paired in a way that the emission wavelength of the donor overlaps with the excitation wavelength of the acceptor, and excitation of the former will stimulate fluorescence of the latter, has been attracting current interest as a means of homogeneous fluoroimmunoassay [5–8]. However, as these methods require at least two antibodies or fragments of an antibody, thus needs a defined condition to conduct an assay with acceptable accuracy. As another type of antibody-based fluorescent biosensor, reagentless-biosensors are developed, which is based on the coupling of environmentally sensitive dye to the residue(s) near the antigen-binding site, e.g., complementarity determining region (CDR) of an antibody [9–11]. In this approach, a fluorophore is introduced at a site that undergoes a change in its environment upon antigen binding. However, there are weaknesses in the method that the variation of suitable dye is limited, and antigens to be measured are limited to proteins.

Recently, our group developed a powerful fluoroimmunosensor called Quenchbody (Q-body), which is a kind of reagentless biosensor but works on a different principle [12]. Q-body works on the mechanism of antigen-dependent removal of quenching effect on popular fluorophores such as carboxyltetramethylrhodamine (TAMRA), which is incorporated to a specific position(s) of a single chain antibody (scFv) or Fab fragment. The primary reason of quenching is photoinduced electron transfer (PET) from conserved tryptophan (Trp) residues in the variable region, and secondarily, the other dye incorporated to the other site [13,14]. Using this Q-body technology, just mixing a Q-body with antigen and measuring the fluorescence intensity enables antigen quantitation. Since no washing step is necessary, it is a remarkably simple and rapid quantification method, still allowing the use of several organic dyes with different colors. Using this innovative biosensor, we could successfully quantify a range of biomolecules including small haptens, peptides, and larger proteins [12–14]. For example, PS82 Q-body was made for the detection of PS82 (vimentin phosphorylation at its 82nd amino acid serine) [13]. At first, we made V_H_-V_L_ type Q-body whose N-terminal region was labeled with a fluorescent dye. We compared its fluorescence intensity in the presence and absence of PS82 peptide, and obtained a modest fluorescence increase of 1.2-fold. As another approach to accomplish better response, we reversed the orientation of V_H_ and V_L_ domains (to V_L_-V_H_) and incorporated an additional fluorophore into the interdomain linker of scFv. As a result, the fluorescence response was successfully enhanced up to 6.7-fold, which gave us an optimistic view of attaining a higher fluorescent response by modulating the structure of Q-body.

**Figure 1.**
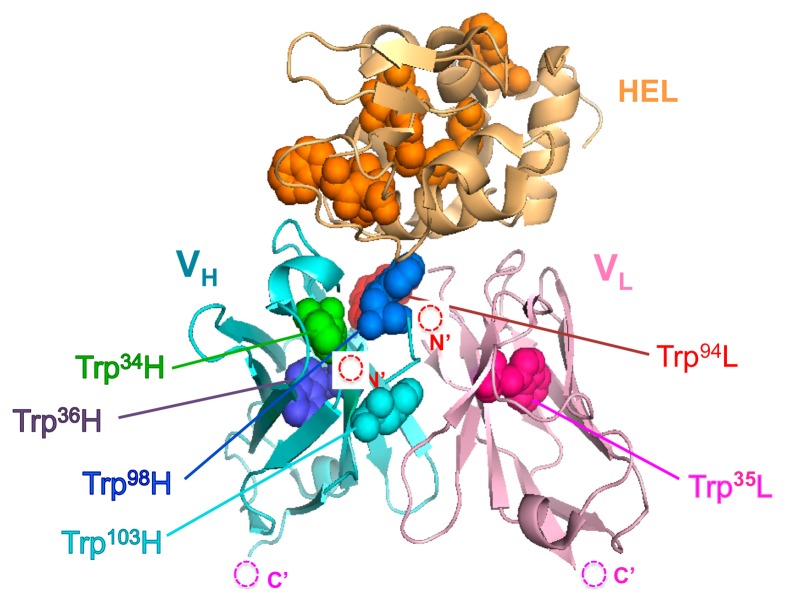
Schematic structure of HyHEL-10 Fv/HEL complex (based on PDB 2dqj). The structure of V_H_, V_L_, and HEL are shown in blue, pink, and orange, respectively. The Trp residues are shown as a space-filling model, with their name for those in the Fv.

Here, we show a more expansive and tangible strategy with a focus on the number and position of fluorophores for the construction of Q-body with high fluorescent response to protein antigens that have intrinsic Trp residues. As a model antibody to evaluate this approach, we used HyHEL-10, which recognizes hen egg white lysozyme (HEL) because its three-dimensional structure has been analyzed by an X-ray crystallography (Figure 1), and the interaction between HyHEL-10 and HEL has been well studied [15,16].

## 2. Experimental Section

### 2.1. Materials

KOD-plus-neo DNA polymerase was from Toyobo (Osaka, Japan). In-Fusion advantage PCR cloning kit was from Takara Bio (Otsu, Japan). QuikChange site-directed mutagenesis kit was from Agilent Technologies (Santa Clara, CA, USA). PureYield plasmid miniprep kit was from Promega (Tokyo, Japan). RYTS cell-free transcription-translation kit was from Roche Diagnostics (Tokyo, Japan). Oxidized glutathione was from Wako pure chemicals (Osaka, Japan). Ni Sepharose High Performance His beads were from GE healthcare (Piscataway, NJ, USA). Nanosep Centrifugal-3 k Ultrafiltration Device was from Pall Corporation (Ann Arbor, MI, USA). Immunoblock was from DS Pharma (Osaka, Japan). Anti-His-HRP antibody was from Qiagen (Hilden, Germany). 3,3′,5,5′-Tetramethylbenzidine (TMBZ), bovine serum albumin (BSA), and HEL were from Sigma (St. Louis, MO, USA). Oligonucleotides were from Operon-Eurofins (Tokyo, Japan). All water used was purified using Milli-Q (Millipore, Tokyo, Japan). Other chemicals and reagents, unless otherwise indicated, were from Sigma or Wako.

### 2.2. Construction of Q-Body Genes

Template genes, primers, and vectors for constructions of each Q-body gene are summarized in Table 1, and the nucleotide sequences of each primer are summarized in Table 2. The details for construction of each gene are as follows: *HL, a V_H_-V_L_ type of HyHEL-10 scFv gene with N-terminal (G_3_S)_2_ and internal (G_4_S)_3_ linkers, is the same gene with pROX-HEL-scFv used on our previous research [12]. *H^(G66^*^)^L, a V_H_-V_L_ type scFv gene whose codon for 66th glycine (G) in the V_L_ was mutated to amber was constructed by QuikChange site-directed mutagenesis according to manufacturer's protocol, by using *HL as a template and HyHEL-L_G66amber and HyHEL-L_G66amber-r as primers. Similarly, *H^(S67^*^)^L, a V_H_-V_L_ type scFv gene whose 67th serine (S) codon in the V_L_ was converted to amber, was constructed with primers HyHEL-L_S67amber and HyHEL-L_S67amber-r.

*LH, a VL-VH type scFv gene incorporated with an amber codon in the N-terminal region of scFv, was amplified by splice overlap extension (SOE) PCR using KOD-plus-neo DNA polymerase and primers VL(HEL)_G3S2Back, VL(HEL)linkFor, LinkVH(HEL)back, and VH(HEL)For, while another VL-VH type scFv gene incorporated with an additional amber codon in the middle of the interdomain linker was amplified with primers LinkVH(HEL)back, VH(HEL)For, LinkAmbBack, and VL(HEL)linkFor. The constructed scFv genes were inserted to DigestedVector (NcoI- and SmaI-digested pROX-FL92.1 amber plasmid [13]) using an In-Fusion PCR cloning kit, resulting in *LH and *L*H, respectively. LH*, a VL-VH type of scFv gene incorporated with an amber codon in the C-terminal region of scFv was amplified with ProX-TTT_HPa+ and ProXtermVkHEL_For and then inserted to AmplifiedVector (amplified pROX-KTM219 gene [12] with primers ProXtermTAGback and ProX-TTT_HPa+_r) using an In-Fusion PCR cloning kit. For *L(G66*)H gene, *H(G66*)L gene was amplified by SOE PCR using primers LinkVH(HEL)back, VH(HEL)for, VL(HEL)_G3S2Back, and VL(HEL)linkFor, and the PCR product was then inserted to DigestedVector using an In-Fusion PCR cloning kit. L*H gene was constructed via QuikChange mutagenesis kit as above by using *L*H as template with primers ProX-TTT_Hpa+ and ProX-TTT_Hpa+_r. The L*H gene was amplified by PCR using L*H as template with primers ProX-TTT_Hpa+ and ProXtermVkHEL_For, and inserted to AmplifiedVector for making L*H* or to DigestedVector for making L(G66*)H using an In-Fusion PCR cloning kit. The L(G66*)*H gene was constructed via QuikChange mutagenesis kit by using L*H as template with primers HyHEL-L_G66amber and HyHEL-L_G66amber-r. L(G66*)H* gene was amplified by PCR using L(G66*)H gene as a template, using primers ProX-TTT_Hpa+ and ProXtermVkHEL_For, and inserted to AmplifiedVector using an In-Fusion PCR cloning kit. Lastly, W35F and W94F genes were constructed by QuikChange mutagenesis as above by using L(G66*)H as template with primers HEL_W35LF and HEL_W35LF_r, and HEL_W94LF and HEL_W94LF_r, respectively. Each of the obtained plasmids was prepared with PureYield plasmid miniprep system, and confirmed the entire coding region sequences.

### 2.3. Synthesis of Q-Bodies

The synthesis of scFv position-specifically incorporated with fluorescence dye was performed using an *E. coli* cell-free transcription-translation system using RYTS kit with 10 mM oxidized glutathione, 150 ng of plasmid DNA, and 400 pmol of amber aminoacyl-tRNA conjugated with TAMRA. The reaction mixture (50 μL) was prepared and incubated at 25 °C for 2 h and subsequently at 4 °C for 16 h.

### 2.4. Purification of Q-Bodies

The synthesized Q-body with C-terminal His-tag was purified using Ni Sepharose High Performance His beads by batch method. First, 100 μL of beads were added to a microtube, and the beads were primed by 400 μL of wash buffer (20 mM phosphate, 0.5 M sodium chloride (NaCl), 60 mM imidazole, 0.1% polyoxyethylene(23)lauryl ether, pH 7.4). Then the reaction mixture, which was diluted in 450 μL of wash buffer, was added to the microtube. After incubation at 25 °C for 15 min, the beads were washed three times with 500 μL of wash buffer by centrifuge (100×g, 1 min, 4 °C). Finally, 400 μL of elution buffer (20 mM phosphate, 0.5 M NaCl, 0.5 M imidazole, 0.1% polyoxyethylene(23)lauryl ether, pH 7.4) was added and incubated at 25 °C for 15 min. After centrifuge (100×g, 1 min, 4 °C), the supernatant was subjected to a Nanosep Centrifugal-3 k Ultrafiltration Device and equilibrated with 500 μL of PBST (10 mM phosphate, 137 mM NaCl, 2.7 mM potassium chloride, 0.05% Tween 20, pH 7.4) two times to exchange buffers. The concentration of Q-body was determined by comparing the fluorescence intensities of a known concentration of TAMRA dye (Anaspec, Fremont, CA, USA) and of the sample under denaturing conditions in 7 M guanidium hydrochloride added with 100 mM DTT, pH 7.4. The resultant Q-body reagent was dispensed at 500 nM and stored at −80 °C.

### 2.5. Enzyme Linked Immunosorbent Assay

After HEL (100 μL, 1 mg/mL in PBS) was immobilized on Falcon 3912 microplate (Tokyo, Japan) for 16 h at 4 °C, the well was filled with 20% Immunoblock in PBS for 2 h at 25 °C, and washed three times with PBST. Subsequently, the purified *HL type Q-body (2 μL) in 100 μL of PBST containing 5% Immunoblock was added and incubated for 2 h at 25 °C. The well was washed three times with PBST and bound Q-body was probed with 4000-fold diluted Penta-His-HRP conjugate in PBST containing 5% Immunoblock for 1 h at 25 °C. The well was washed three times with PBST, and developed with 100 μL of substrate solution (100 μg/mL TMBZ and 0.04 μL/mL hydrogen peroxide, in 100 mM sodium acetate, pH 6.0). After incubation for 5 min, the reaction was stopped with 50 μL of 10% sulfuric acid, and the absorbance was read at 450 nm with a reference at 655 nm using a microplate reader Model 680 (Bio-Rad, Tokyo, Japan). As a control, nothing was immobilized on microplate, and the same procedure was performed.

### 2.6. Fluorescence Measurements

Each Q-body (500 nM, 7.5 μL) was diluted in 250 μL of PBST containing 1% BSA, and HEL was added by titration in a 5 × 5 mm quarts cell (Starna, Atascadero, CA, USA). After each addition, the solution was incubated for 2 min at 25 °C prior to the spectral measurements. The fluorescence intensity was measured using a fluorescence spectrophotometer Model FP-8500 (JASCO, Tokyo, Japan) with excitation at 546 nm and emission from 562 to 662 nm. The excitation and emission slit widths were set to 5.0 nm. Fluorescence intensities at the maximum emission wavelength (580 nm) and the normalized fluorescence intensity of each sample based on the fluorescence intensity at zero dose were plotted. Dose-response curves were fitted using Kaleida Graph 4.1 (Synergy Software, Reading, PA, USA) and the EC_50_ values were calculated from the curve fitting to a 4-parameter logistic equation.

## 3. Results and Discussion

### 3.1. Construction of HEL Q-Body

**Figure 2.**
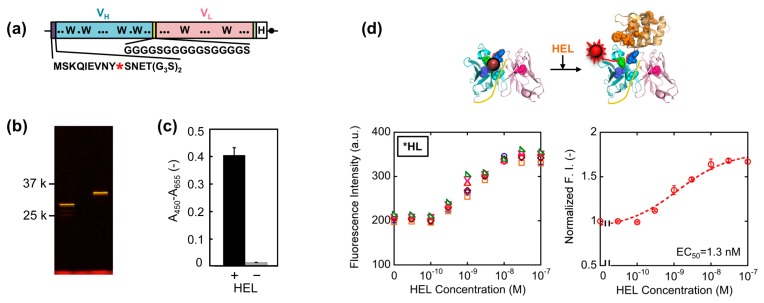
(**a**) Scheme of *HL type HEL Q-body gene. Asterisk denotes an amber codon; (**b**) Fluorescence image of SDS-PAGE for the expressed *HL type HEL Q-body in non-reducing (left) and reducing (right) conditions; (**c**) Specific binding of HEL Q-body to HEL, probed by ELISA. Error bars represent ±1 standard deviation (SD) (n = 3); (**d**) Conceptual drawing of the de-quenching of the Q-body (upper), and titration curves of the fluorescence intensity (lower left) and normalized fluorescence intensity (lower right) of *HL type Q-body. Error bars represent ±1 SD (n = 6).

First, we constructed a V_H_-V_L_ type HEL Q-body gene by linking the 3′ of V_H_ and 5′ of V_L_ of HyHEL-10 gene with a flexible (G_4_S)_3_ linker, preceded by a ProX-tag and (G_3_S)_2_ linker and followed by a His-tag (Figure 2a). Next, we synthesized the protein, and site-specifically incorporated fluorescence dye TAMRA as an unnatural amino acid into the N-terminal region of scFv via amber codon in ProX-tag using a cell-free transcription-translation system [17]. After purification using His-tag, the purified Q-body was resolved by SDS-PAGE and detected by fluorescence imaging of the gel (Figure 2b). By comparing the bands of reduced sample and of non-reduced sample, it is verified that the protein was successfully expressed with folding. Furthermore, it is also confirmed from the result of SDS-PAGE that TAMRA was successfully incorporated into the protein and the unbound dye was clearly eliminated by purification. Next, we investigated the antigen binding activity of the Q-body by ELISA. Significant signal was observed for the wells immobilized with HEL, while negligible signal was observed for the wells without HEL (Figure 2c).

This result clearly shows the binding activity of HEL Q-body to HEL even after incorporation of fluorescent dye into the protein. Finally, the fluorescence intensity of the Q-body was measured in the presence of varied concentration of HEL. The fluorescence intensity was increased upon addition of HEL, indicating that the initially quenched fluorescence was de-quenched by the addition of antigen. The fluorescence titration curve clearly indicates HEL concentration-dependent increase in fluorescence intensity, with the maximal fluorescence response of 1.7-fold (70% increase) (Figure 2d). Its EC_50_ value was 1.3 nM, which was somewhat lower than the previously reported EC_50_ value of this Q-body (7.5 nM) [12]. Compared with previous Q-body, minor modifications in the protein expression condition such as reaction temperature and addition of oxidized glutathione (10 mM) were introduced, which could result in the somewhat improved response.

### 3.2. Increased Response by Optimization of Q-Body Construct

**Figure 3.**
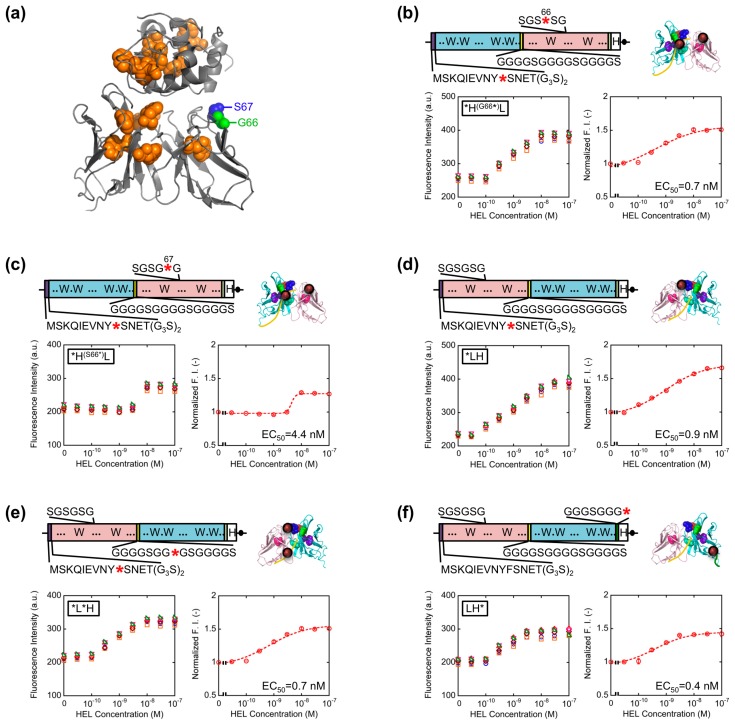
(**a**) The position of the 66th Gly and the 67th Ser residues of HyHEL-10 V_L_; (**b**–**f**) Schematic presentations of the gene (upper left), schemetic structure of Q-body in the absence of antigen (upper right), titration curves of the fluorescence intensity (lower left) and normalized fluorescence intensity (lower right) of each Q-body. Error bars represent ±1 SD (n = 6).

**Figure 4.**
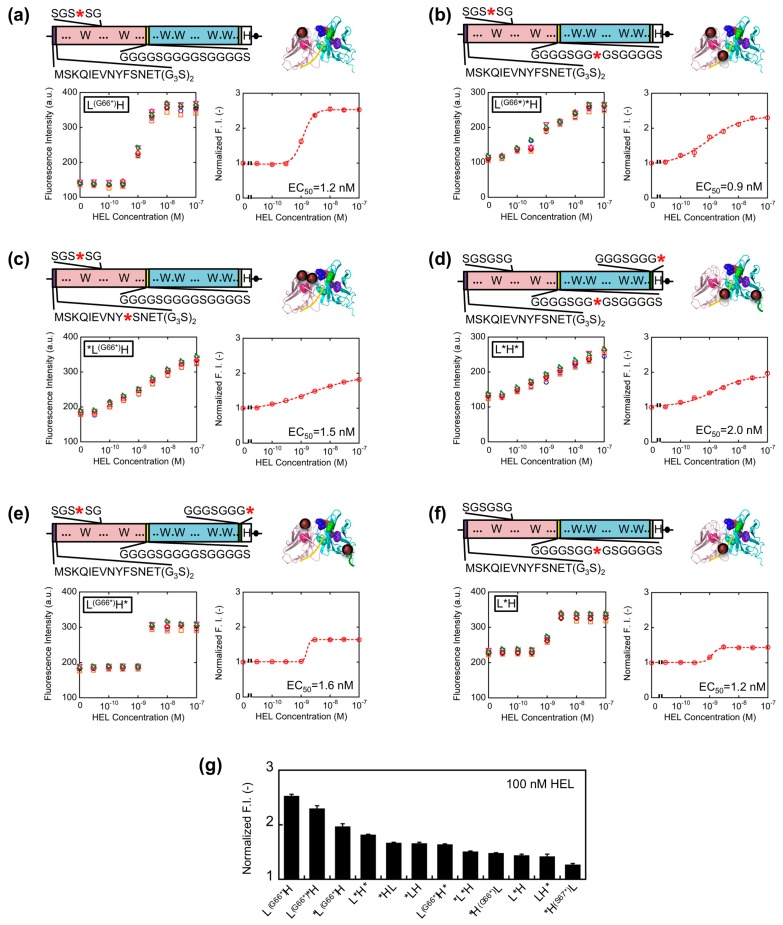
(**a**–**f**) Schematic presentations of each Q-body gene (upper left) and of possible location of the dye(s) on the Q-body in the absence of antigen, and corresponding titration curves (lower). Error bars represent ±1 SD (n = 6); (**g**) Comparison of the fluorescence responses of Q-body upon addition of 100 nM HEL. Error bars represent 1 SD (n = 6).

The result obtained so far corresponded well with the result of our previous HEL Q-body. This time, in order to obtain Q-bodies with higher response, we further examined other types of Q-bodies incorporating several new strategic constructs focusing on the number and position of fluorophores. The constructs made are as follows: (1) *H^(G66^*^)^L and *H^(S67^*^)^L types; we noticed that the 62–67th residues of the V_L_ domain is SGSGSG, which is a GS-rich sequence that possibly allows incorporation of large unnatural amino acids [13]. In addition, from the three-dimensional structure of HyHEL-10 Fv, there is a V_L_ core Trp residue (Trp35L) near this non-complementarity determining region (CDR) loop sequence (Figure 3a). From these facts, we expected a possibility of making a novel Q-body in which a fluorolabeled amino acid is incorporated in this loop. Hence, we changed the Gly66L or Ser67L to an amber codon by site-directed mutagenesis (Figure 3b,c). Since this loop sequence is not CDR, there is little concern on the prevention of antigen binding; (2) *LH type; the domain orientation of scFv was reversed to V_L_-V_H_, and a fluorophore was introduced to the ProX-tag at the N-terminal region (Figure 3d); (3) *L*H type; To *LH, an additional fluorophore was incorporated to the middle of interdomain (G_4_S)_3_ linker (Figure 3e); (4) LH* type; From the fact that there are Trp residues not only in the scFv but also in the antigen HEL, another construct based on LH with a dye incorporated to the site far from the Trp residues in HEL to avoid the quenching by HEL. An amber codon was attached after a (G_3_S)_2_ linker at the C-terminal of scFv (Figure 3f). The five types of Q-bodies were expressed by using a cell-free transcription-translation system in a high-throughput manner in spite of large number of samples. As a result, all the five Q-bodies showed HEL concentration dependent fluorescence intensity with high sensitivity. In particular, the *LH type Q-body showed the highest response among the six Q-bodies including *HL type. Actually, we previously thought that the response of *LH type might be lower than *HL type because there were more Trp residues in the V_H_ domain (four) than in the V_L_ domain (two). However, *LH type Q-body unexpectedly showed higher response than *HL type, implying the importance of quenching by the Trp residues in the antigen HEL as well as those in the scFv. In other words, in the case of *HL type Q-body, the fluorophore incorporated to the N-terminal region was difficult to be de-quenched after adding HEL, because there are many Trp residues in HEL near the epitope of V_H_ domain. On the other hand, since the Trp residues are more distant from the V_L_ epitope, the fluorophore attached to V_L_ could be more easily de-quenched.

Since somewhat enhanced fluorescent response was shown for V_L_-V_H_ type Q-bodies than V_H_-V_L_ types, we further extended the variation of V_L_-V_H_ type Q-bodies on the basis of the results obtained above. To this end, L^(G66^*^)^*H, *L^(G66^*^)^H, L^(G66^*^)^H, L*H*, L^(G66^*^)^H*, and L*H type genes were constructed (Figure 4a–f). For the mutation in SGSGSG sequence, the codon for G66L was changed to an amber codon because *H^(G66^*^)^L showed higher response than *H^(S67^*^)^L. As a result, the fluorescence intensity of all the Q-bodies increased in a HEL concentration-dependent manner, and four of them showed higher response than *HL (Figure 4g). The result thus indicates a successful construction of superior Q-bodies by adjusting the number and position of fluorophores. Among these twelve kinds of Q-bodies, L^(G66^*^)^H showed the highest response of 2.5-fold (150% increase). In addition, the slope of the titration curve of L^(G66^*^)^H was steeper than that of L^(G66^*^)^H* and *L^(G66^*^)^H, even though the structures and EC_50_ values were similar.

It is possible that the two fluorophores stayed close to each other as in the cases of dual labeled Q-body, where PET and/or quenching H-dimer formation [18] between the two fluorophores might happen. If H-dimer formation happens, de-quenching is harder to be induced, resulting in the necessity of the higher concentration of antigen for complete de-quenching. As for the reason of lower response for L^(G66^*^)^H*, one of the dyes incorporated to the C-terminal region might be difficult to be quenched by the Trp residues in scFv due to their distances, and such unquenched fluorescence will increase the background signal, resulting in the lower response.

### 3.3. Involvement of Internal Trp Residues in the Quenching of TAMRA at Position 66L

**Figure 5.**
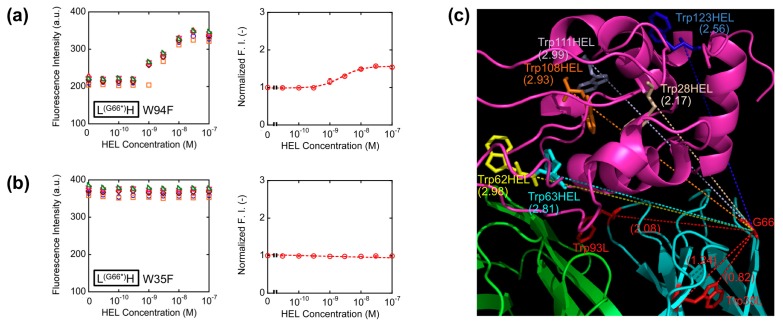
Titration curves for W94F (**a**) and W35F (**b**) mutant of L^(G66^*^)^H Q-body. Error bars represent ±1 SD (n = 6); (**c**) Distance from the position 66 to Trp94L, Trp35L, or to the Trp residues in HEL. The Cα-Cα Trp residues to the position 66 shown in parentheses were measured using PyMOL. The unit of distance is nm.

With the aim of elucidating the involvement of Trp residues in the observed fluorescence quenching of the loop labeled Q-bodies, we decided to make Trp-to-Phe mutants. This time, we introduced mutations to the two Trp residues in the V_L_ of L^(G66^*^)^H (W35F and W94F), and compared their responses with that of the wild-type L^(G66^*^)^H (Figure 5a,b). As a result, the fluorescence enhancement observed upon antigen addition was 1.5-fold for W94F, while no apparent antigen dependency was observed for W35F. Obviously, both responded less than the wild-type (2.5-fold). It is worth noting that the mutant of the Trp residue nearest to the dye (W35F) showed markedly reduced response, which suggests deep quenching of the wild-type by Trp35L. Since Trp94L is farther from the position 66 (the Cα-Cα distance is 2.08 nm) than Trp35L (Cα-Cα: 1.24 nm, while the nearest neighbor distance is 0.82 nm) (Figure 5c), it will not quench the fluorophore so much, thus resulting in the lower decrease of W94F response.

These results are in accordance with our hypothesis that the antigen-dependent fluorescence of TAMRA at position 66L is also due to PET by internal Trp residues, not due to the change of dye environment. Furthermore, since the position 66 is far from each of six Trp residues in HEL (Figure 5c), the possibility of quenching by Trp residues of HEL is lower than other constructs, for example *HL, resulting the better response of L^(G66^*^)^H.

## 4. Conclusions

Here we reported a novel strategy to increase the response of anti-protein Q-body. Namely, a GS-rich flexible loop region in the V_L_ that is not used as a CDR was successfully utilized as a position to introduce TAMRA dye by using a position-specific *in vitro* labeling system. The Q-body labeled at this position showed the highest response, which was attributable to the quenching by internal Trp residues and its antigen-dependent release. Moreover, this position locates far from any Trp residues in the antigen, while it is close to the Trp35 in the V_L_ core. According to the Abysis database [19], the amino acid sequence preceding 66L is highly conserved. Probably, such location is ideal for attaining maximal quenching in the absence of antigen and its maximal antigen-dependent release.

## Figures and Tables

**Table 1.** Template and primers used for the construction of each Q-body gene.

**Q-Body Gene**	**Insert**		**Vector ^(a)^**	**Cloning Method**

	**Template**	**Primers**		
*H ^(G66^*^)^L	*HL	HyHEL-L_G66amber, HyHEL-L_G66amber-r	-	QuikChange
*H^(S67^*^)^L	*HL	HyHEL-L_S67amber, HyHEL-L_S67amber-r	-	QuikChange
*LH	*HL	VL(HEL)_G3S2Back, VH(HEL)linkFor, LinkVH(HEL)back, VL(HEL)For	DigestedVector	In-Fusion
*L*H	*HL	LinkVH(HEL)back, VH(HEL)For, LinkAmbBack, VL(HEL)linkFor	DigestedVector	In-Fusion
LH*	*LH	ProX-TTT_HPa+, ProXtermVkHEL_For	AmplifiedVector	In-Fusion
*L^(G66^*^)^H	*H^(G66^*^)^L	LinkVH(HEL)back, VH(HEL)for, VL(HEL)_G3S2Back, VL(HEL)linkFor	DigestedVector	In-Fusion
L*H	*L*H	ProX-TTT_HPa+, ProX-TTT_HPa+_r	-	QuikChange
L*H*	L*H	ProX-TTT_HPa+, ProXtermVkHEL_For	DigestedVector	In-Fusion
L^(G66^*^)^H	L*H	ProX-TTT_HPa+, ProXtermVkHEL_For	DigestedVector	In-Fusion
L^(G66^*^)^*H	L*H	HyHEL-L_G66amber, HyHEL-L_G66amber-r	-	QuikChange
L^(G66^*^)^H*	L^(G66^*^)^H	ProX-TTT_HPa+, ProXtermVkHEL_For	AmplifiedVector	In-Fusion
W35F	L^(G66^*^)^H	HEL_W35LF, HEL_W35LF_r	-	QuikChange
W94F	L^(G66^*^)^H	HEL_W94LF, HEL_W94LF_r	-	QuikChange

(a) DigestedVector: *Nco*I- and *Sma*I-digested pROX-FL92.1 amber plasmid; AmplifiedVector: Amplified pROX-KTM219 gene with primers ProXtermTAGback and ProX-TTT_HPa+_r.

**Table 2.** Nucleotide sequences of the primers used.

**Primer Name**	**Nucleotide Sequence (5′-3′)**
HyHEL-L_G66amber	cctccaggttcagtggcagttagtcagggacagatttcac
HyHEL-L_G66amber_r	gtgaaatctgtccctgactaactgccactgaacctggagg
HyHEL-L_S67amber	ggttcagtggcagtggataggggacagatttcactc
HyHEL-L_S67amber_r	gagtgaaatctgtcccctatccactgccactgaacc
VL(HEL)_G3S2Back	tagtctaatgagaccggtggcggttcaggtggcggttcagacattgtgctgaccc
VL(HEL)linkFor	tccgcctgaaccgcctccaccccgtttgatttccagcttgg
LinkVH(HEL)back	ggcggttcaggcggaggtggctctggcggtggcgatctgaggtgcagctgcagg
VH(HEL)For	atgagaaccccccccgctcgagacggtgaccagggtccc
LinkAmbBack	ggcggttcaggcggatagggctctggcggtggc
ProX-TTT_HPa+	ctaaacaaatcgaagttaacttttctaatgagacc
ProXtermVkHEL_For	ctcccgagcccccgccggaggagacggtgaccagg
ProX-TTT_HPa+_r	ggtctcattagaaaagttaacttcgatttgtttag
ProXtermTAGback	cggcgggggctcgggaggtggatagggggggggttctcatcatc
HEL_W35LF	caacagagtaacagcttcccgtacacgttcggag
HEL_W35LF_r	ctccgaacgtgtacgggaagctgttactctgttg
HEL_W94LF	gtattggcaacaacctacacttctatcaacaaaaatcacatgagtc
HEL_W94LF_r	gactcatgtgatttttgttgatagaagtgtaggttgttgccaatac
